# A Latent Profile Analysis of Psychotic Experiences, Non-psychotic Symptoms, Suicidal Ideation and Underlying Mechanisms in a Sample of Adolescents From the General Population

**DOI:** 10.3389/fpsyt.2022.926556

**Published:** 2022-06-27

**Authors:** Álvaro I. Langer, Klaas Wardenaar, Johanna T. W. Wigman, José Luis Ulloa, Daniel Núñez

**Affiliations:** ^1^Mind-Body Lab, Institute of Psychological Studies, Faculty of Medicine, Universidad Austral de Chile, Valdivia, Chile; ^2^Agencia Nacional de Investigaciòn y Desarrollo (ANID), Millennium Nucleus to Improve the Mental Health of Adolescents and Youths, Imhay, Santiago, Chile; ^3^Associative Research Program, Center of Cognitive Sciences, Faculty of Psychology, Universidad de Talca, Talca, Chile; ^4^Department of Psychiatry, Interdisciplinary Centre for Psychopathology and Emotion Regulation, University Medical Center Groningen, Groningen, Netherlands

**Keywords:** psychotic experiences, psychopathology risk, regulatory strategies, adolescence, latent profile analysis

## Abstract

Psychotic experiences (PEs) are prevalent in the general population, particularly in adolescents. PEs are associated with various negative outcomes such as psychotic, depressive, anxiety and post-traumatic stress disorders and suicidal behavior. Recent studies in the general population have suggested that what makes PEs relevant is not so much the experiences *per se*, but their association with non-psychotic comorbidity and other transdiagnostic domains. Thus, there is a need for a better understanding of how PEs exist in a larger psychopathological context in adolescents. In the present study we aimed to explore this, using latent profile analysis (LPA) to identify different patterns in which PEs, psychiatric symptoms and psychological processes co-occur. LPA was conducted using data from an adolescent general population subsample (*n* = 335) with PEs. We conducted LPA, using measures of PEs, psychiatric symptoms and behaviors (depression, anxiety post-traumatic stress disorder and suicidal behavior) and cognitive and affective processes of entrapment/defeat and emotional regulation as manifest variables. We found that the best fit was obtained with a four-class solution that distinguished primarily between different levels of overall severity: “low symptomatology” (19.1%), “mild-moderate symptomatology” (39.4%), “moderate symptomatology” (33.7%); “high symptomatology” (7.8%). Levels of depression, post-traumatic stress symptoms and defeat/entrapment were most differentiated between classes. The high symptomatology group showed the highest scores in all psychiatric symptoms suicidal ideation, and emotional/cognitive domains, except in cognitive reappraisal. This group also showed the highest usage of emotional suppression. Our results suggest that the assessment of mental health risk in adolescents should be aware that PEs exist in a broad context of other domains of psychopathology and transdiagnostic cognitive and affective processes.

## Introduction

Psychotic experiences (PEs) are prevalent in the general population (1), particularly in adolescents (2). In addition to indexing risk for later psychotic disorder (3), PEs are associated with various other negative outcomes such as depressive, anxiety (4–6), and post-traumatic stress disorders (PTSD) (7–9), as well as with suicidal behavior (10). PEs are also linked to both higher psychiatric treatment utilization (11), and greater public mental health burden (12). Despite its clinical relevance and its association with poorer treatment outcomes (13), PEs are frequently undetected by clinicians (14). The clinical significance of PEs in childhood and adolescence and its role in the transition to psychopathology is not fully understood yet (15, 16). Although PEs have been suggested as predictors of mental disorders in the general population (17) and psychotic symptoms in youth at clinical high risk for psychosis (18), recent studies in the general population have highlighted that what makes such experiences psychopathologically relevant is not the experiences *per se*, but rather their co-occurrence with other domains of non-psychotic comorbidity (e.g., depression, anxiety and PTSD) (9, 19) as well as the presence of other vulnerability factors such as a history of adverse childhood events (20). In addition, as different subdimensions of PEs seem to be differentially associated with concurrent psychiatric comorbidity (21–23), and because some PEs might be healthy coping mechanisms helping to maintain mental functioning (24), these associations should be assessed according to specific PE dimensions (18).

Taking a more transdiagnostic perspective of psychopathology (25, 26), one way to approach this is to analyze PEs in conjunction with other symptoms and vulnerability factors in clinical and non-clinical populations within the framework of the extended psychosis phenotype model (27). One technique that allows for this is latent profile analysis (LPA), a categorical latent variable modeling approach (28, 29) allowing for classifications of individuals into latent subgroups based on their scoring patterns on a range of manifest variables. LPA results can be useful to develop and incorporate data-driven typologies in empirical research (30). LPA studies with adolescents presenting with PEs are scarce (31) but may yield important new insights. In a clinical high risk population, Healy et al. (32) evaluated PEs in association with depression and found three classes. When comparing these classes using neurocognitive measures, social cognition and functioning measures, they observed that the participants belonging to class 1 (low PEs and depression scores) showed intact neurocognitive and social functioning but that participants of class 3 (higher PEs and depression scores) showed higher neurocognitive and social cognitive impairment. In a non-clinical population of adolescents, Fonseca-Pedrero et al. (33) assessed schizotypal traits, and found four latent profiles that were subsequently compared on emotional and behavioral difficulties, cognitive functioning, suicide ideation and bipolar-like experiences. They observed that the group with high scores in all schizotypy domains showed the higher scores on mental health difficulties, suicide ideation, bipolar-like experiences and PEs. Finally, Lucas-Molina et al. (31) assessed PEs, schizotypal traits, and bipolar-like experiences in Spanish adolescents, and also found 4 clusters. The two groups with respectively moderate and high psychotic liability scored significantly higher on mental health difficulties and negative affect, and lower on positive affect and well-being.

Overall, these studies suggest the existence of three to four subgroups with different levels of psychopathological risk and that this risk is associated with affective (bipolar-like and depressive symptoms), and neurocognitive and social functioning levels, which in turn are linked to the intensity of PEs and schizotypal latent traits (31–33).

To further our understanding of the manifestation of psychopathology in adolescents, several studies have suggested that assessing underlying cognitive and affective processes in conjunction with psychiatric symptoms may be fruitful (34–36). Examples of such processes are defeat and entrapment, which are considered transdiagnostic mechanisms present in different psychopathological conditions and behaviors (37), including psychosis (38, 39) and suicidal behavior (40). Earlier work on linking such processes with psychopathology has found that increased defeat and entrapment are associated with higher intensity of PEs in adolescents (41). Another relevant mechanism is emotion regulation, defined as conscious and non-conscious strategies used to increase, maintain, or decrease one or more components of an emotional response (42). Two specific emotion regulation strategies that have been associated with PEs (43) and psychotic disorders (44) are emotional suppression and cognitive reappraisal. Specifically, it has been shown that adolescents endorsing PEs use more emotional suppression and less cognitive reappraisal (45), which is in turn associated with higher comorbid symptomatology (46). However, the evidence of emotional difficulties in psychotic disorders remains mixed (25, 47).

In the present study, we sought to explore latent profiles reflecting subgroups of individuals with different patterns of PEs, psychiatric symptoms and psychological processes in a sample of adolescents from the general population endorsing PEs (*n* = 335). Taking a more transdiagnostic perspective to psychopathology (26), we identified different subgroups of adolescents based on their profile on multiple psychopathological domains and several transdiagnostic emotional/cognitive processes that underlie multiple psychiatric disorders. In line with the literature (24), we analyzed different sub-dimensions of PEs. We hypothesized that the LPA would reveal latent classes with different symptom profiles, mostly differentiated by the severity of symptoms, the intensity of PEs and by different levels of underlying transdiagnostic processes. Moreover, we hypothesized that the groups with higher psychopathological scores will show a higher usage of maladaptive transdiagnostic strategies.

## Methods

### Participants

We conducted a cross-sectional study with 1,599 adolescents recruited between April and September 2019 in 11 public secondary schools in Chile, using a convenience sample. Voluntary participation by both the students and their parents and signed informed consent was required. To select a subsample of participants, we used two inclusion criteria. First, we included participants with PEs as indicated by a score >1.47 on the Community Assessment of Psychic Experiences CAPE-P15 (48, 49). Second, we only included participants with > points on at least one item of the CAPE-P15. Our final sample consisted of 335 adolescents (mean age= 15.62; SD= 1.39, women = 65.1%).

### Measures

#### Psychotic Experiences

We used the CAPE-P15 (49), a 15-item self-report questionnaire. In the current version, responses to items range from 1 (never) to 5 (very often). This version was validated in Chile in a sample of adolescents (50). The scale assesses three domains: paranoid ideation (PI, 5 items), bizarre experiences (BE, 7 items), and perceptual anomalies (PA, 3 items). Sum scores can range from 15 to 75. Higher scores indicate higher severity of PE. In our sample, the reliability of the scale was good (Cronbach's α= 0.94; McDonald's Omega = 0.884 = 0.81).

#### Anxiety Symptoms

We used the Generalized Anxiety Disorder 7-item scale (GAD-7) (51), a 7-item self-report questionnaire with responses ranging from 0 (not at all) to 3 (nearly every day). Total scores can range from 0 to 21 points. Higher scores indicate higher severity of anxiety. The GAD-7 was tested specifically in adolescents and previously used in a Chilean study (52). In our sample, Cronbach's α was 0.90 and McDonald's ω was 0.86.

#### Depressive Symptoms

We used the Patient Health Questionnaire-9 (PHQ-9) (53), a 9-item self-report questionnaire with responses ranging from 0 (not at all) to 3 (nearly every day). Total scores can range from 0 to 27. Higher scores indicate higher severity of anxiety. We used the validated version for Chilean adolescents (52). In our sample, Cronbach's alpha (α) was 0.90, and McDonald's Omega Coefficient (ω) was 0.81.

#### Post-traumatic Stress Symptoms

We used the Brief Post-Traumatic Stress Disorder scale (BPTSD) (54), a 8 item self-report scale with responses ranging from 1 (never) to 5 (very often). It encompasses two factors; PTSD (5 items) and self-organization disturbances DSO (3 items). Higher scores indicate higher severity of PTSD. This version was validated in Chile in a sample of adolescents (52). In our sample, the reliability of the complete instrument was good (Cronbach's α = 0.88, McDonald's Omega = 0.88).

#### Suicidal Ideation

We used seven items of the Columbia Suicide Severity Rating Scale (C-SSRS) (55), adapted for use as a self-report questionnaire in a Chilean sample of adolescents (56). Each item is scored dichotomously (0–1). The severity of SI was rated on a 7-point ordinal scale in which 1 = wish to be dead, 2 = nonspecific active suicidal thoughts, 3 = thoughts about how to commit suicide, 4= suicidal thoughts and intentions, 5 = suicidal thought with a detailed plan, 6 = intentions to conduct plan, 7 = prior behaviors or planning acts to commit suicide. Participants with positive scores in the three first items (over 3 points) were classified as elevated risk of suicidal ideation. Participants were asked whether thoughts happened: ever in life (SIL) and/or during the last month (SIM). We only reported SIL because there were few reports of SIM. In our sample, α was 0.90 and ω was 0.81.

#### Defeat and Entrapment

We used the Short Defeat and Entrapment Scale (SDES) (57). It comprises eight items with a 5-point response scale ranging from 1 (never) to 5 (very often). Four items assess defeat, defined as the perception of a failed struggle, feelings of powerlessness, and a sense of losing social status or missing personal goals. Four items assess entrapment, defined as the feelings of being threatened or involved in a stressful, unpleasant state or situation which one cannot escape because of internal or external circumstances. Total scores for each scale range from 4 to 20. Higher scores indicate a higher use of defeat and entrapment. In our sample, α and ω values for the Defeat scale were 0.93 and 0.89 respectively, while for the Entrapment scale they were 0.86 and 0.84, respectively.

#### Emotional Regulation

We used the Emotional Regulation Questionnaire (ERQ) (58). It comprises 10 items with a 7-point scale from 1 (totally disagree) to 7 (totally agree). Four items assess emotional suppression (SUPR) and six items address cognitive reappraisal (REAPR). Higher scores mean a higher usage of each emotional regulation strategy. Total scores for the SUPR and REAPR scales range from 6 to 42 and from 4 to 28 respectively. In our sample, the α and ω values for the SUPR scale were 0.61 and 0.62 respectively, while for the REAPR scale they were 0.78 and 0.79.

### Procedure

Participants completed online questionnaires, administered in school computer laboratories by members of the research group. Respondents had approximately 30 min to answer the questionnaires. All subjects provided written informed consent and the research protocol was approved by the Ethics Committee of the Universidad de Talca.

### Statistical Analysis

First, we calculated the descriptive statistics of the questionnaires assessing symptoms, suicidal ideation, defeat/entrapment and emotional regulation. Second, we computed Pearson correlation coefficients to investigate the interrelationships between the variables. Third, to identify latent classes based on the manifest variables, we conducted a Latent Profile Analysis (LPA), using all above described scales as manifest variables. LPA is a type of latent variable model that explains heterogeneity in a sample by identification of two or more latent classes, based on subjects' scores on a range of continuous input variables (59, 60). LPA was run using the “mclust” package (61) in R (version 4.0.3; 57). The “mclust” package was used to estimate LPA models with 1–9 classes with four different model configurations. The latter differ in the way the manifest-variables variances and covariances are fixed, constrained and/or freely estimated across classes and differ in terms of complexity. These model configurations are: (1) the manifest variable covariances fixed to zero and their variances constrained to be equal across classes (equal volume, equal shape [and undefined orientation]; EEI), (2) the covariances matrix is estimated and constrained to be equal across classes (equal volume, equal shape, and equal orientation; EEE), (3) the manifest variable covariances are fixed to zero and their variances freely estimated in each class (varying volume, varying shape [and undefined orientation]; VVI), and (4) the covariances matrix is estimated freely for each class (varying volume, varying shape, varying orientation; VVV). In the analyses, each of the combinations of class-number and model configuration was estimated. In total, 32 models were fit (1–9 classes ^*^ 4 configurations) using Expectation Maximization (EM). The best fitting model was selected based on the optimal Bayesian Information Criterion (BIC). The “mclust” uses an approach to calculate the BIC that yields values where the highest BIC indicates the best model (62). After identification of the optimal model, each subject was allocated to a class based on their highest class probability.

Finally, we compared the mean manifest variable scores between the latent classes using independent samples *t*-tests. An alpha of 0.05 was used and Bonferroni correction was applied to correct for multiple comparisons.

## Results

### Descriptive and Correlational Analyses

Descriptive statistics and correlations are shown in Supplementary Table 1. The correlations between all study variables were positive, low-strong, and statistically significant (*p* < 0.001).

### Latent Profile Analysis

Of the fitted LPA models, the 4-class model with an EEE configuration was selected as the optimal model based on the BIC values (Supplementary Table 2). In this model, the mean scores varied across latent classes, but the variable covariance matrix was equal across classes. The mean classification uncertainty was 0.085 and mean class-probabilities ranged from 0.88 to 0.97 across classes, indicating considerable class-allocation certainty.

**Figure 1:**
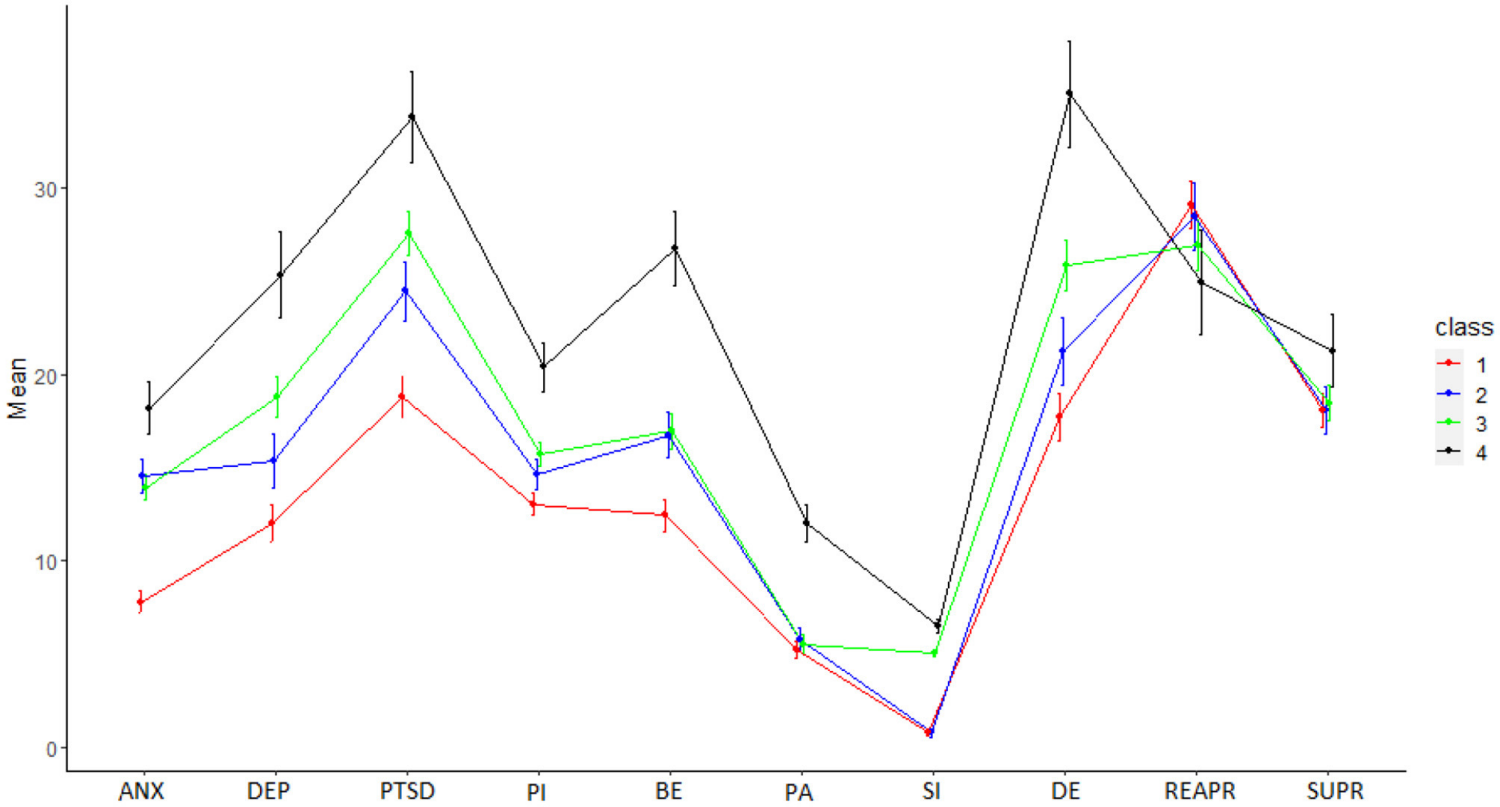
Latent Profile Analysis (LPA): Four latent profile solutions. ANX, Anxiety; DEP, Depression; PTSD, Post Traumatic Stress Disorder; PI, Paranoid Ideation; BE, Bizarre Experiences; PA, Perceptual Anomalies; SI, Suicidal Ideation; DE, Defeat and Entrapment; REAPR, cognitive reappraisal; SUPR emotional suppression.

The visual inspection of the class-specific mean scores on the manifest variables (Figure 1) showed that the four classes differed primarily in terms of the severity levels of the manifest variables. Therefore, we labeled the classes as follows: “low symptomatology” (*n* = 64; 19.1%), “mild” (*n* = 132; 39.4%), “moderate symptomatology” (*n* = 113; 33.7%), and “high symptomatology” (*n* = 26; 7.8%).

Finally, Supplementary Table 3 shows the comparison of manifest variable scores among latent classes. The variables showing differences among all clusters were depression, PTSD, and defeat/entrapment. The “high symptomatology group” showed the highest scores in all PEs and also in suicidal ideation. Finally, this group presented the highest scores in emotional suppression.

## Discussion

We identified specific psychopathological profiles of adolescents from the general population enriched for psychotic experiences, based on a latent profile analysis (LPA) on two broad domains: first, psychopathological symptoms (depressive, anxiety and post-traumatic stress symptoms; suicidal ideation and psychotic experiences) and second, emotional/cognitive processes (emotional suppression, cognitive reappraisal, defeat/entrapment). In line with prior research (4, 7, 63), we found that these two domains are correlated. Our results showed that the best fit was obtained with a four-class solution, which we labeled as representing “low symptomatology” (19.1%), “mild-moderate symptomatology” (39.4%), “moderate symptomatology” (33.7%); “high symptomatology” (7.76%). Thus, our findings support our first hypothesis stating that LPA would reveal latent classes with different symptom profiles. Our second hypothesis was partially supported, because just defeat/entrapment presented differences among groups, and emotional suppression was only higher in the “high symptomatology group”. Overall, these results are in accordance with prior research (64), suggesting that PEs, psychiatric symptoms and cognitive/affective regulatory processes co-exist as a continuum of mental distress and that severity of non-PE symptoms and related processes/experiences meaningfully differentiated subgroups within the sample.

The first class is characterized by low intensity of PEs and low scores in all assessed psychiatric symptoms and emotional/cognitive processes. This class is similar to the non-risk class found by Lucas-Molina et al. (31) and the low schizotypy class identified by Fonseca-Pedrero et al. (33). However, our “low class” presents a lower percentage of participants grouped in this cluster compared with the abovementioned studies (44.2 and 62.4% respectively). This difference might be due to the fact we used a sample of adolescents who effectively endorsed psychotic experiences. Then, it is possible to argue that it seems logical to expect that only a relatively small percentage of our sample will present low levels of symptoms.

The “mild” and “moderate” classes did not differ in severity of anxiety symptoms or PEs. These classes did present intermediate scores for paranoid ideation and bizarre experiences, but not for perceptual anomalies. The “moderate class” did show higher symptom scores of depression, posttraumatic stress symptoms, suicidal ideation as well as defeat/entrapment. The abovementioned pattern observed for PEs in these two groups is different from Luca-Molina et al. (31) observed in their classes with low and intermediate schizotypal risk (classes 2 and 3 respectively). Their class called “high reality distortion experiences” displayed higher scores in nearly all domains, with the exception of anhedonia, schizotypy, psychotic and bipolar dimensions. Additionally, this group showed higher levels of mental health difficulties and lower well-being. This suggests that the more clinicopathological configuration observed in the “moderate” group might not be explained by the presence of PEs *per se*. This supports recent research highlighting the relevance of non-psychotic disorders when assessing psychopathological risk in adolescents (19).

The class we labeled as the “high-risk” class showed the highest scores in all psychiatric symptoms, suicidal ideation and emotional/cognitive domains, except in cognitive reappraisal. The relatively small percentage observed for this class (7.76%) is similar to the lower prevalence previously reported for those classes with the most severe and persistent PE in the general population (64–67), and resembles the group labeled as “high psychosis liability” by Luca-Molina et al. (31). The finding of this class supports literature showing that a higher intensity of PEs is related to a higher number of overall symptoms (68), suicidal related behaviors (69), psychological distress (70), and emotional dysregulation processes (46, 71) in some individuals.

It is worth mentioning that our approach differs from previous LPA studies analyzing EPs, which first identified PE classes and subsequently compared them to other psychopathological variables (31, 33). By contrast, in accordance with evidence showing that the mere presence of PEs does not discriminate between individuals with and without mental disorders (72), we sought to differentiate between profiles that explicitly combine PEs and other variables, which could then result in e.g., classes that have similar levels of PEs, but different score profiles on the other variables. Thus, our approach is closer to those modeling the co-occurrence of mental disorders were PEs reflect a unitary latent continuum of mental distress associated with common mental disorders, suicidal related behaviors and poor treatment outcomes (64). Therefore, direct comparisons should be interpreted with caution.

We observed that depression, PTSD symptoms and defeat/entrapment were the variables showing significant differences among classes. Thus, our finding supports the suggestion that depression is one of the most relevant variables to index the clinicopathological risk of the PEs in adolescents (19, 65). To our knowledge, this is the first LPA study including PTSD symptoms in an attempt to cluster adolescents reporting PEs. Our results align with recent research using different methodologies revealing association between PEs and trauma in adolescents (20), highlighting that PTSD symptoms are relevant in differentiating psychopathological profiles in adolescents. Additionally, defeat/entrapment has not been included in previous LPA models and our study is, to our knowledge, the first to look at their co-expression with psychopathological symptoms in adolescents with PEs. Our results showed that these processes are differentially associated with different combinations of co-occurring symptoms. This supports its relevance as a transdiagnostic process for several mental disorders (73), and suggests that they may underlie the differential levels of distress experienced by participants belonging to each cluster.

Whereas, the emotion regulation process of cognitive reappraisal seems to be used in the same way by all groups, emotional suppression was more used by participants belonging to the high-risk class. Thus, the prominent presence of emotional suppression in this class could be a potential process increasing the distress experienced by individuals with high levels of psychopathological symptoms. This is in line with previous findings showing that it is a maladaptive strategy associated with psychotic symptoms (44, 47) and that emotional regulation difficulties play a translating role from childhood trauma into distressing PEs in later life (43).

This work also has limitations. The sample size is relatively small, but it fits in the range of sample sizes for which most fit indices in latent class techniques have been shown to work well (n~300–1,000) (74). The cross-sectional design does not allow for inferring causal relationships among variables. Although the measurement scales are designed for general and subclinical populations, the variables may have presented some variability restriction in their lower range (i.e., floor effect), which could limit statistical power. Moreover, other variables associated with PEs, such as adverse childhood experiences (75), attachment styles (76) and cognitive functioning (77) were not studied. In addition, although our work was based on the premise of extended psychosis phenotype, it has been found that PEs may not be the same experience in clinical and non-clinical populations (78); thus, cautious interpretations must be considered to apply these results to more clinical populations. Future research tackling these limitations is required.

In conclusion, based on a broad range of transdiagnostic variables that reflected multiple domains of psychopathology as well as several transdiagnostic cognitive/affective processes, we identified four homogeneous subgroups in a sample of adolescents from the general population enriched for PEs. Our results provided new insights on specific differential configurations of psychopathological vulnerability in this group. Thus, to accurately screen out low risk PEs could allow destigmatizing these experiences. We confirmed that depression is relevant to differentiate regarding vulnerability among adolescents with PE and added new evidence revealing that PTSD symptoms play also a prominent role. Moreover, we support that maladaptive cognitive and emotional regulatory strategies should also be considered as vulnerability proneness to psychopathology.

## Data Availability Statement

The raw data supporting the conclusions of this article will be made available by the authors, without undue reservation.

## Ethics Statement

The studies involving human participants were reviewed and approved by Comité Ético Científico, Universidad de Talca. Written informed consent to participate in this study was provided by the participants' legal guardian/next of kin.

## Author Contributions

DN designed the study and directed its implementation, did the literature search, and wrote the manuscript. ÁL wrote the manuscript. KW and JU performed the statistical analyses. JW reviewed the manuscript and revised it critically for intellectual content. All authors contributed to the article and approved the submitted version.

## Funding

DN, ÁL, and JU were supported by ANID—Millennium Science Initiative Program (NCS2021_081), and Programa de Investigación Asociativa (PIA) en Ciencias Cognitivas, Facultad de Psicología, Universidad de Talca (RU-158-2019). ÁL was partially funded by ANID—Millennium Science Initiative Program— ICS13_005.

## Conflict of Interest

The authors declare that the research was conducted in the absence of any commercial or financial relationships that could be construed as a potential conflict of interest.

## Publisher's Note

All claims expressed in this article are solely those of the authors and do not necessarily represent those of their affiliated organizations, or those of the publisher, the editors and the reviewers. Any product that may be evaluated in this article, or claim that may be made by its manufacturer, is not guaranteed or endorsed by the publisher.

## Supplementary Material

Supplementary Table 1 Descriptive statistics and bivariate correlations. ^*^*p* < 0.05; ^**^*p* < 0.01; ^***^*p* < 0.001.

Supplementary Table 2 Bayesian Information Criterion (BIC). EEI, Equal volume, Equal shape, Undefined orientation; EEE, Equal volume, Equal shape, Equal orientation; VVI, Varying volume, Varying shape, Undefined orientation; VVV, Varying volume, Varying shape, Varying orientation.

Supplementary Table 3 Comparison of manifest variable scores among latent classes.
